# Unusual positron emission tomography findings in pulmonary amyloidosis: a case report

**DOI:** 10.1186/1749-8090-1-32

**Published:** 2006-10-05

**Authors:** Sumit Yadav, Sanjay Sharma, Ian Gilfillan

**Affiliations:** 1 Department of Cardiothoracic Surgery, Fremantle Hospital, Fremantle, WA (6160), Australia

## Abstract

Positron emission tomography (PET) has come to play an increasingly important role in the evaluation of pulmonary lesions, which are suspicious of malignancy. As is true for other techniques, PET gives false positive and negative results. We report a case of pulmonary amyloidosis with multiple pulmonary nodules showing different uptake of 18 F-fluorodeoxyglucose (FDG) on PET. There are limitations of specificity of FDG-PET in characterising pulmonary nodules and it is important to confirm a suspected malignancy with histology before potentially curative treatment is undertaken.

## Background

PET imaging is a technology that is used to identify focal areas of increased cellular metabolism. FDG is an excellent tracer for detecting malignant disease because of high glucose metabolism observed in cancer cells. With this technique whole body image can be created to accurately identify malignant primary neoplasm and metastatic disease.

For pulmonary lesions FDG-PET has been used to differentiate malignant from benign lesions and to stage and follow patients with lung cancer. PET has demonstrated sensitivity and specificity rates of 80–90% compared to 50–60% for CT [1]. Low-grade malignant lesions such as carcinoids and localised broncho-alveolar carcinomas have shown low to intermediate glucose metabolism and negative PET images [2]. On other hand, there have been reports of false positive PET in metabolically active benign lesions like tuberculous granulomas, coccidiomycosis and aspergillosis [3,5]. There have been anecdotal case reports of false positive PET in pulmonary amyloidosis [5,6].

Amyloidosis is a disorder in which insoluble protein fibres become deposited in tissues and organs. The exact mechanism that causes amyloidosis is unknown. However, primary amyloidosis appears to be related to abnormal production of amino globulins by plasma cells whereas secondary amyloidosis is associated with the presence of chronic inflammation of infectious disease. A variety of inflammatory and infectious disorders can result in increased FDG activity. Gallium 67, an inflammatory seeking agent, has been reported to have shown intense uptake in amyloid goitre as a result of inflammatory action. It is conceivable that inflammatory action caused by amyloid lesion result in increased FDG uptake[7]. What makes our case interesting is the fact that in the same patient different amyloid nodules had different uptake of FDG on PET.

## Case report

**Figure 1:**
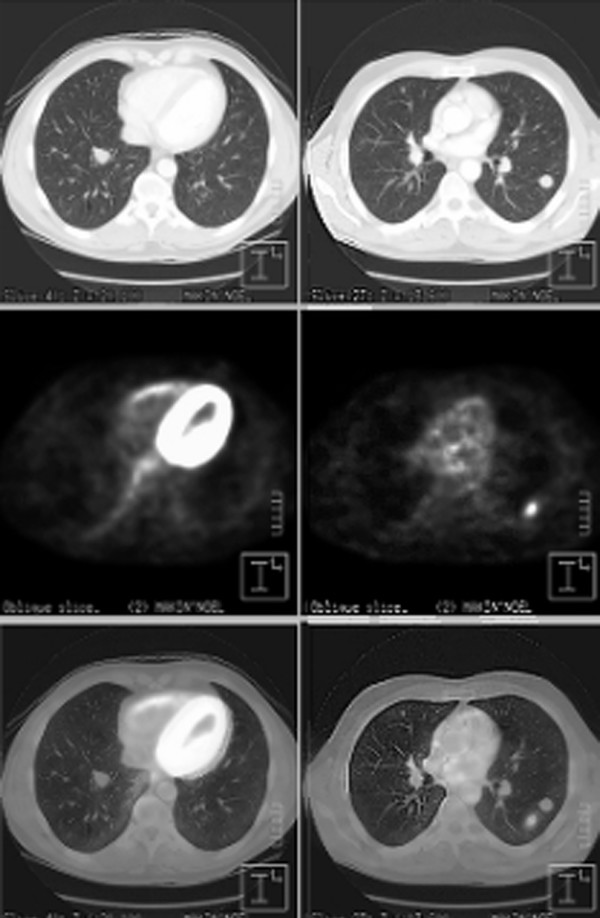
*Top panel: *Computed tomography scan showing multiple rounded nodules in the right and left lung fields. *Middle panel: *Corresponding sections on PET scan. *Bottom panel: Superimposition *of PET scan on CT scan shows that the intense uptake on PET scan corresponds to the lesion in the left lower lobe on CT scan. The rest of the lesions in lung fields do not show any uptake on PET scan.

**Figure 2:**
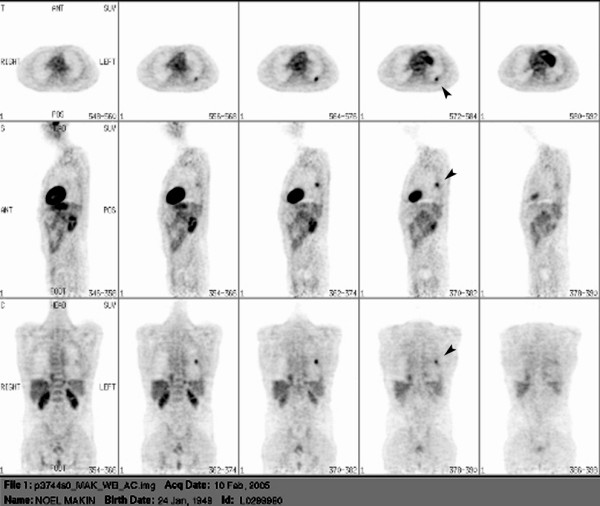
PET scan demonstrating a focus of intense FDG activity (arrow mark) in the left lung field.

A 55-year-old farmer was referred to our hospital for further evaluation of multiple pulmonary nodules detected on his chest x-ray for his chronic cough. He was a smoker with a history of asbestos exposure. His physical examination and laboratory findings were unremarkable. A computed tomography (CT) scan of the chest revealed multiple bilateral focal pulmonary lesions varying in size from 1 to 2.5 cm with no evidence of lymphadenopathy. The largest nodule measuring 2.5 × 2.5-cm was noticed in the apical segment of the left lower lobe (Figure 1, *top panel*). CT findings were suspicious of multiple pulmonary metastasis. He had FNA of one of the peripheral nodules on the left side under CT guidance and the microscopy was suggestive of nodular amyloidosis. To rule out malignancy in the rest of the nodules he underwent an FDG-PET scan (Figure 2). On PET scan there was intense focal uptake in the left lower lobe which corresponded with the dominant pulmonary nodule on CT scan and the degree of activity was highly suspicious of neoplastic origin (Figure 1, *bottom panel*). There was no other metabolically active lesion seen in the lungs that would correspond with the nodular opacities identified on CT. Though some of the nodules were below the size resolution of PET and remained uncharacterised, however, others were large and in the absence of significant uptake these were thought to be benign on PET scan. Given the dangers of reporting benign disease on FNA, it was thought prudent to confirm the diagnosis with excision biopsy. Surgical exploration revealed a well-demarcated palpable mass in the apical segment of the left lower lobe. There was another smaller nodule present in the left upper lobe. Both of these were wedged out and sent for frozen section. The histopathology findings were consistent with pulmonary amyloidosis in both the nodules. Afterwards, the patient was investigated for evidence of myeloma or plasmacytoma. All subsequent investigations including bone scan, bone marrow aspiration trephine were normal and ruled out secondary amyloidosis.

## Conclusion

This case emphasises the limitations of specificity of FDG-PET in characterising pulmonary nodules and the importance of confirming suspected malignancy with histology before potentially curative treatment is undertaken. Further studies for mechanism of such false positive results on PET scan are warranted.

## Competing interests

The author(s) declare that they have no competing interests.

## Authors' contributions

Sumit Yadav – sequence alignment and drafted the manuscript.

Sanjay Sharma – helped to draft the manuscript.

Ian Gilfillan – conceived of the case report.
